# Significant reduction in bacterial shedding and improvement in milk production in dairy farms after the use of a new inactivated paratuberculosis vaccine in a field trial

**DOI:** 10.1186/1756-0500-2-233

**Published:** 2009-11-22

**Authors:** Ramon A Juste, Marta Alonso-Hearn, Elena Molina, Marivi Geijo, Patricia Vazquez, Iker A Sevilla, Joseba M Garrido

**Affiliations:** 1 Department of Animal Health, NEIKER-Tecnalia, Berreaga 1 E-48160 Derio, Bizkaia, Spain

## Abstract

**Background:**

Paratuberculosis vaccination has been in use in some regions for many decades, but results have not been widely spread. A new *Mycobacterium avium *subsp. *paratuberculosis *(MAP) killed vaccine was studied in relationship with its effects on fecal shedding and milk production in four farms while other two were kept as controls submitted to a test and cull scheme.

**Findings:**

Fecal detection (n = 1829) and milking records (n = 2413) have been analyzed after two (5 herds) and four (1 herd) years of the beginning of the intervention. Shedder prevalence was reduced by 100% in three of the four vaccinated farms, 68% in the total of vaccinated animals and 46% in the two control farms. Total amount of MAP shed was reduced 77% in the vaccinated farms and 94% in the control farms. Overall milk production increased up to 3.9% after vaccination, while there was no significant difference in production after intervention in the non-vaccinated farms.

**Conclusion:**

MAP shedding reduction can be quickly accomplished both by vaccination and by testing and culling. However, vaccination appears to be a less expensive and more sustainable strategy since it required one single intervention and was also associated with an increase in milk production.

## Background

Paratuberculosis or Johne's disease eradication programs based on the detection and culling of infected animals (testing and culling, T&C) have been relatively unsuccessful due to the low sensitivity of diagnostic tests and unending expenses for detection of infection in individual animals. Even though vaccination has been successfully used for over 30 years in the US and in the UK as well as in other countries, scientific reports on its efficacy are old and scarce in spite of having shown to yield higher benefit/cost ratios than T&C strategies [1,2].

Near eradication of bovine tuberculosis in the Basque Country, as well as high prevalence of clinical cases of paratuberculosis in some farms, led the local Animal Health Authorities to support a vaccination trial in farms with a history of heavy clinical incidence. This trial was implemented as a field assay to test the efficacy of a new paratuberculosis vaccine specifically designed for use in cattle and that is based in whole cell heat-killed MAP (Silirum^®^, CZ Veterinaria, S.A., Porriño, Spain) in an oil adjuvant. The objective of the present report is to evaluate its performance on MAP fecal excretion as an indicator of epidemiologic efficacy, to assess any possible therapeutic effects of vaccination and to estimate direct benefits in milk production that could facilitate the spread of this control strategy by encouraging farmers to use of the vaccination as a control strategy at their own expense. The follow up is scheduled to last five years in each herd, and therefore the results presented here are a preliminary assessment.

## Findings

### MAP detection in feces

**Figure 1:**
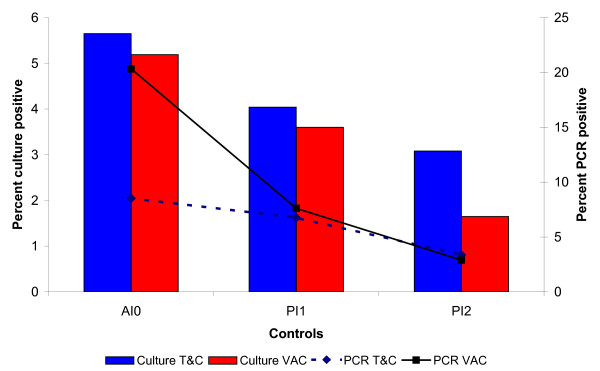
**Prevalence of cows excreting MAP in their feces at each fecal control as measured by isolation and by IS900 PCR**. AI0: Before intervention, PI1: First post-intervention control; PI2: Second control post-intervention. Culture T&C: Results of fecal culture in the two herds without vaccination. Culture VAC: Results of fecal culture in the four herds with vaccination. PCR T&C: Fecal IS900 PCR positive results in the two herds without vaccination. PCR VAC: Fecal IS900 PCR positive results in the four herds with vaccination.

**Figure 2:**
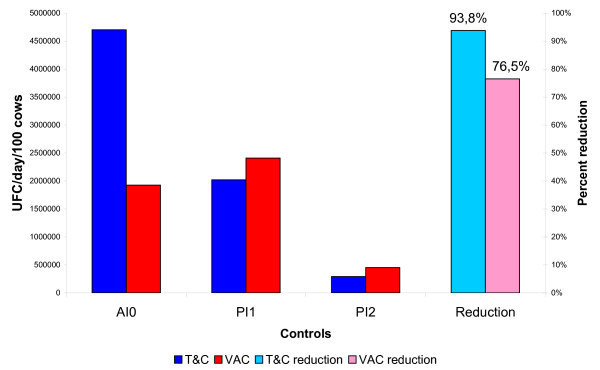
**Evolution of estimated amount of daily bacterial shedding per cow according to the strategy**. Although in the first years T&C appears to be substantially more efficient, vaccination still seems to reduce shedding by three fourths. AI0: before intervention, PI1: First post-intervention control; PI2: Second control post-intervention; Reduction: Percent of reduction in the estimated amount of bacteria shed from the initial to the last control. Notice that this approach shows a slower reduction with vaccination than when only frequency of shedders is considered.

Fecal shedding frequencies by PCR and by isolation before intervention and at each annual sampling are shown in table 1 and figure 1. Significant reduction in fecal shedding across herds ranged from 71% to 90% with PCR, and from 75% to 100% with culture. Globally, vaccination seemed to induce greater reductions (86% and 68% for PCR and culture, respectively) than T&C (60% and 46% for PCR and culture, respectively. This relationship seemed to be inverted when the amounts of shedding were compared (Figure 2). For this variable, T&C (4,702,381 CFU/day/100 cows to 292,308 CFU/day/100 cows) yielded a reduction of 94% versus a 76% associated to vaccination (1,925,926 CFU/day/100 cows to 452,675 CFU/day/100 cows) (Figure 2).

**Table 1:** Frequency of fecal shedding at different time point as detected by IS900 PCR and isolation and rate of reduction between pre-intervention sampling and last control.

IS900-PCR		AI0		PI1		PI2		PI4					
Herd		n	%	n	%	n	%	n	%	Herd shedding reduction	P	Strategy reduction	p
T&C	HER	196	7.65	195	4.62	182	2.20		-	**71.1%**	0.0126	**60.4%**	0.0039
	URI	144	9.72	176	9.09	143	4.90		-	**49.6%**	0.0890		
	AGE	76	17.11	76	2.63	79	2.53		-	**85.2%**	0.0019		
VAC	END	0	-	0	-	64	3.13	71	0.0	100.0%*	0.2229		
	SAG	46	32.56	48	0.00	34	5.88		-	**81.9%**	0.0036	**85.8%**	<0.0001
	SAL	63	15.87	60	20.00	65	1.54		-	**90.3%**	0.0037		

MAP isolation		AI0		PI1		PI2		PI4					
Herd		n	%	n	%	n	%			**Herd shedding reduction**	P	Strategy reduction	P

T&C	HER	193	6.74	195	3.59	182	1.65			**75.4%**	0.0127	**45.5%**	0.0760
	URI	143	4.20	176	4.55	143	4.90			**-16.7%**	0.7140		
	AGE	76	2.63	76	2.63	79	0.00	-	-	**100.0%**	0.2388		
VAC	END	88	7.95	50	10.00	64	3.13	71	0.0	100.0%*	0.0142		
	SAG	46	0	48	0.00	38	5.26			**-**	0.1981	**68.2%**	0.0243
	SAL	63	7.94	60	1.67	65	0.00			**100.0%**	0.0266		

T&C: Testing and culling; VAC: Vaccination; HER, URI, AGE, END, SAG, SAL: Herd codes; AI0: Ante-intervention sampling; PI1: First post-intervention sampling; PI2: Second post-intervention sampling (includes 48 months control for farm END); PI4: Fourth post-intervention sampling (corresponds to 60 months post-intervention only for END). Reduction is calculated as the quotient of the difference in proportions to the initial proportion. * Herd reduction calculated at the last control for END. p: Fisher exact test probability of the difference being due to chance.

### Milk production

A total of 2762 lactations, of which 2413 were complete 305-day standard lactations (SL), were used for the analysis of variance. The overall model including parity, strategy (testing and culling or vaccination) and intervention (ante- and post-intervention) and all their interactions was highly significant on standard and real lactation milk production, days of lactation and average daily production (ADP). Only the models for SL and ADP as dependent variables had an R^2 ^value greater than 0.10 and thus only these were retained for the final analysis (Table 2). The SL model, with an R^2 ^value of 0.1792, showed that the strongest effects were those of parity and strategy (p < 0.0001) and the interaction between strategy and intervention (p = 0.0259). Upon marginal means comparison, it appeared that there was no significant difference between ante- and post-intervention SL in the T&C herds (-186.87 kg; p = 0.2318), while there was a difference of 257.13 kg in the vaccinated ones (p = 0.0376) (Table 2). No other effect or interaction had a p value below 0.10. Regarding the ADP, the only significant effects were again those of the strategy and parity interaction between strategy and ante- or post-intervention periods (p = 0.0035). The differences between ante- and post-intervention ADP were -0.54 kg (p = 0.2079) and 1.08 kg (p = 0.0020) respectively for T&C and vaccination. The latter accounted for a difference of 329.4 kg in SL. The cows in the T&C strategy showed higher variability in the differences in production, with a significant decrease (p = 0.0336 SL; p = 0.0030 ADP) for the second calving. The vaccinated herds showed always a positive balance between ante- and post-intervention milking records, with significant increases in production for first (p = 0.0759, N.S. SL; p = 0.0247 ADP) and third (p = 0.0422; p = 0.2627, N.S.) calving.

**Table 2:** Milk production

Dependent variable	Strategy	Vaccination	No. of records	Marginal mean (kg)	Difference	P
	T&C	Ante-intervention	1040	10374.96	-186.87	0.2318
		Post-intervention	242	10288.09	(1.80%)	
Standard lactation	VAC	Ante-intervention	767	9420.51	257.13	0.0379
		Post-intervention	364	9677.64	(2.73%)	
	T&C	Ante-intervention	1040	31.55	-0.21	0.7486
Average daily production		Post-intervention	232	31.34	(0.67%)	
	VAC	Ante-intervention	767	29.27	1.15	0.0016
		Post-intervention	364	30.42	(3.92%)	

* First sampling date was set as the ante/post-vaccination threshold.

## Discussion

The results presented here show that both T&C and vaccination had a significant effect on the bacteriologic variables relevant to the epidemiology of paratuberculosis. Even though the timing and magnitude of the reductions were not the same in all the farms, the overall effect in terms of frequency of shedders and of estimated amount of bacteria excreted were greatly reduced both by T&C and by vaccination. It is somewhat surprising that so good results were observed in such a short period of time. These observations are in agreement with most works on paratuberculosis vaccination [3-15].

Regarding milk production, in this study, vaccination performed better than T&C. This is in agreement with reports on paratuberculosis related milk losses [1,16] even though in a study vaccination had a slight negative effect on non-infected animals that was compensated by the large effects on milk production of advanced cases of paratuberculosis[9].

An important issue in this study is that the controls have not been taken from matched individuals in the same herd, but that each farm has been on one strategy. This was a difficult decision taken at the beginning of the study in order to avoid the influence of treatment on one group affecting the other in the long period of follow up imposed by the slow infection characteristic of paratuberculosis. This, in addition to the need of farmers of an immediate and readily visible solution to their paratuberculosis problem, as well as the management difficulties of herds with mixed strategies, made it more advisable to use a before-/after- comparison strategy in spite of its limitations in order to attribute the effects solely to vaccination.

Our results, obtained in over 80% of cases from animals vaccinated after at least three months of life in its infected farm, show that there might be therapeutic effects related to the pathogenesis-modifying effects of vaccination that were already observed twenty years go by Benedictus et al[5]. This implies that vaccination could not only provide an immediate relief to farms affected by a heavy incidence of paratuberculosis, but would allow to foresee a completely new strategy for treatment of human IBD if its paratuberculosis etiology is demonstrated and accepted by the gastroenterological community.

In conclusion, the results presented here show that similar levels of paratuberculosis control can be achieved in a short period of time both by T&C and by vaccinating with the new killed vaccine, at least in some farms. If eradication is ruled out as the failure of long term strategies shows[17,18], vaccination stands out as the most economically efficient strategy at similar or better epidemiologic performances with T&C[2]. Since it involves protecting animals instead of killing the infected ones, vaccination is also a more sustainable strategy for paratuberculosis control than T&C.

## Materials and methods

### Farm selection

Six Holstein Friesian herds of the Basque Country with a history of clinical paratuberculosis whose owners were willing to collaborate were selected. The annual incidence of paratuberculosis clinical cases in these herds ranged from 2 to 10%. They were officially free of bovine tuberculosis for, at least, the last 5 years as determined by regular intradermal tuberculin test. The first herd (END) to enter the study was vaccinated in January 2003 and the other three (AGE, SAG, SAL) were vaccinated in March 2006. Two herds of similar characteristics were kept as controls submitted to a test and cull strategy without vaccinating any animals. This strategy consisted in recommending the farmers to cull animals with an indirect ELISA or fecal PCR positive result. The two non-vaccinated herds were sampled for the first time in May, 2006 and this date was set as the threshold for intervention temporal comparisons.

### Vaccine administration

One ml of the Silirum^® ^MAP vaccine (CZ Veterinaria, S.A., Porriño, Spain), was administered subcutaneously into the dewlap of all animals of all ages present in the farm at the moment of joining the trial, and then to all the new 1-2 month old replacer female calves. This resulted in that over 80% of the post-vaccination observations were from animals vaccinated when older than 3 months. Each dose contained 2.5 mg of heat-killed 316F MAP strain plus an oil adjuvant and thiomersal as preservative.

Experimental vaccination was carried out according to the Spanish legislation. It was authorized by the competent local Animal Health and Animal Experimentation authority (Animal Health Service of the Diputación Foral de Gipuzkoa and Diputación Foral de Bizkaia, respectively for the vaccinated and non-vaccinated herds), the Spanish drug registration authority (Agencia Española de Medicamentos y Productos Sanitarios as AEM n° 107/ECV) and well as by the central Animal Health authority (Spanish Ministry of Agriculture, Fisheries and Food, currently Ministerio de Medio Ambiente y Medio Rural y Marino).

### Fecal sampling

Feces were collected from the rectum of the cows older than 24 months on the ante-intervention (AI0) control at the time of reading the intradermal test and at yearly intervals afterwards. They were stored at 4°C and processed within 48 h of arrival to the laboratory for DNA isolation and for fecal culture.

### Amplification of MAP DNA from fecal samples

Isolation of MAP DNA from fecal samples was performed using the Adiapure MAP DNA extraction and purification kit (Adiagene, Saint Brieuc, France). Two μl of DNA solution were tested with a commercial qualitative Real-Time PCR kit based on the amplification of a segment of the MAP IS900 sequence (Adiagene, Saint Brieuc, France) according to the manufacturer instructions. For each set of reactions, positive (MAP DNA from an ATCC19698 culture) and negative (no DNA) controls were included. The amplifications were performed in an ABI Prism type 7000 (Applied Biosystems, Foster City, CA, US) thermal cycler under the following conditions: 2 min at 50°C, 10 min at 95°C and 45 cycles of 15 s at 95°C and 60 s at 60°C. Results were read as positive when the reaction showed a typical amplification curve and a Ct value below 40.

### Detection of MAP by fecal culture

Culture of MAP from fecal samples was performed as previously described [19] on home-made Herrold's Egg Yolk medium (HEYM) and in Löwenstein-Jensen medium (L-J) (Difco, Detroit, Michigan, US), both supplemented with mycobactin J (Allied Monitor, Fayette, Missouri, US)[20]. Tubes were observed every 4 weeks and considered negative if after 20 weeks no bacterial growth was observed. Samples were considered positive if 1 or more colonies forming units (CFU) were morphologically identified as MAP in 1 or more culture tubes. Animals were classified as low shedders (< 10 CFU; estimated average 2 CFU/tube), medium shedders (10 to 50 CFU; estimated average 20 CFU/tube) and heavy shedders (> 50 CFU; estimated average 200 CFU/tube). Colony identity was confirmed by PCR amplification of the IS900 MAP insertion sequence, as described above.

### Milk production

Records of individual cow production kept by the Basque Federation of Friesian Breeders (EFRIFE) were kindly provided by M. Eugenia Amenabar. They included date of birth, date of calving, monthly milk production record, days of lactation and real and 305 days-standard milk production per lactation. The records corresponding to years 2000 to 2007 were used representing up to 5 years before and after intervention.

### Statistical analysis

The frequency of shedders before and after intervention according the herd strategy (vaccination or test and cull) were compared using the SAS statistical package Fisher exact test of the FREQ procedure (SAS Institute Inc., Cary, NC 27513, USA). A quantitative estimate of overall bacterial contamination by fecal shedding was made according to the quantitative equivalences defined above for each level of colony counts. After natural logarithm transformation the results were submitted to analysis of variance and least square means comparison with the GLM procedure taking as independent variables the type of intervention, the time after intervention and their interaction.

For milk production, the SAS GLM procedure was used for analysis of variance and least square mean comparison of real lactation, days of lactation, standard 305 days lactation kg of milk (SL) and average daily production (kg of milk divided by actual number of days in lactation, ADP) as dependent variables. In this model, strategy, intervention (ante- and post-intervention date) and parity were used as independent variables. All the least square means where compared using the Student's t test with the Tukey adjustment for multiple pair-wise comparisons.

Reductions and increases were calculated as the quotient of the difference between the compared and the reference level mean or frequency to the reference level mean or frequency.

## Competing interests

RAJ had expenses paid by a company for giving a conference, attending a meeting and making an expert report for registration of a vaccine against paratuberculosis two years ago. RAJ, MVG and JGA own a small participation of an equity that supports a small company that is a spin-off of NEIKER in a project for the development of an improved vaccine for paratuberculosis.

## Authors' contributions

RAJ designed the experiment, performed the statistical analysis and wrote the final version of this report. JMG contributed to the experimental design and oversaw the farms follow up. MAH helped in the writing. EMO, MGE, PVA, ISE and JMG participated in the sampling and processing of the fecal samples. All the authors read the final version of this manuscript.
